# Supplementary figures and images for: GALDAR: A genetically encoded galactose sensor for visualizing sugar metabolism in vivo
Source: PLoS Biol. 2024 Mar 19;22(3):e3002549. doi: 10.1371/journal.pbio.3002549 (PMC10950222; doi:10.1371/journal.pbio.3002549)

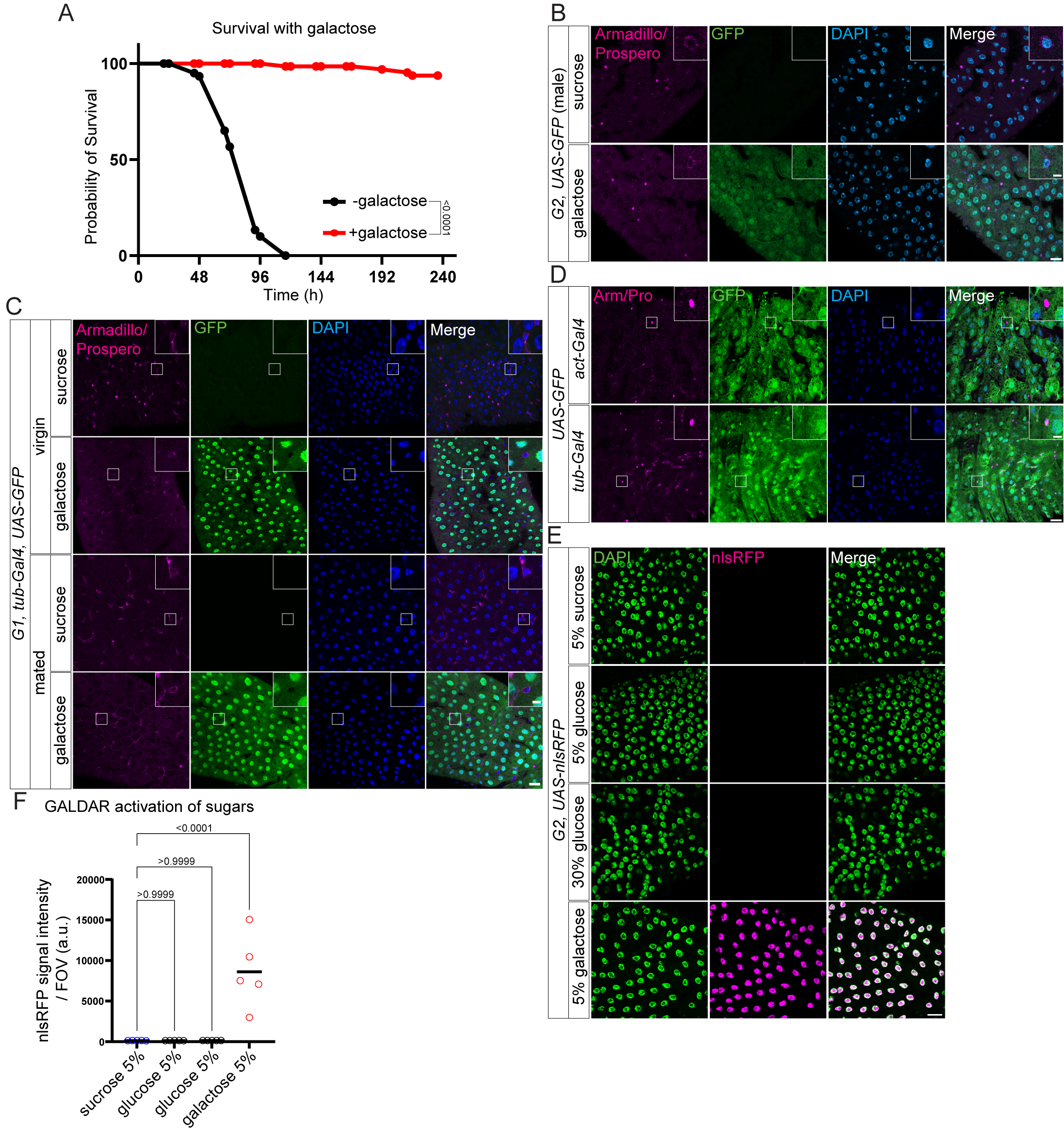

Supplement: S1 Fig — (A) Survival of adult male OregonR flies fed without (black line, n = 62), or with 5% galactose (red line, n = 76) addition to a basal agar medium containing 0.5% sucrose. Logrank (Mantel–Cox) test. (B) Confocal images of R2 regions of midguts of male G2, UAS-GFP flies fed with 0% (top) or 5% (bottom) galactose addition for 3d. ISCs are highlighted in small panels. (C) Confocal images of R2 regions of midguts of virgin (top) or mated (bottom) female G1, tub-Gal4, UAS-GFP flies fed with 0% or 5% galactose addition for 3d. ISCs are highlighted in small panels. (D) Confocal images of R2 regions of act-Gal4 (top) or tub-Gal4 (bottom) midguts with UAS-GFP. EECs are highlighted in small panels. Note the lack of GFP in act-Gal4, UAS-GFP EECs. (E) Confocal images of midgut R2 regions of G2, virgin female G2, UAS-nlsRFP flies fed with 5% sucrose, 5% glucose, 30% glucose, and 5% galactose (DAPI in green, nlsRFP in magenta). (F) Quantification of nlsRFP fluorescence intensity per field of view (n = 5 for each condition), ANOVA with multiple comparison test. Data are representative of at least 3 independent experiments. S1 Data provides the source data used for all graphs and statistical analyses. Scale bars, 20 μm in large, 5 μm in small panels in all figures. (TIF) [file pbio.3002549.s001.tif]

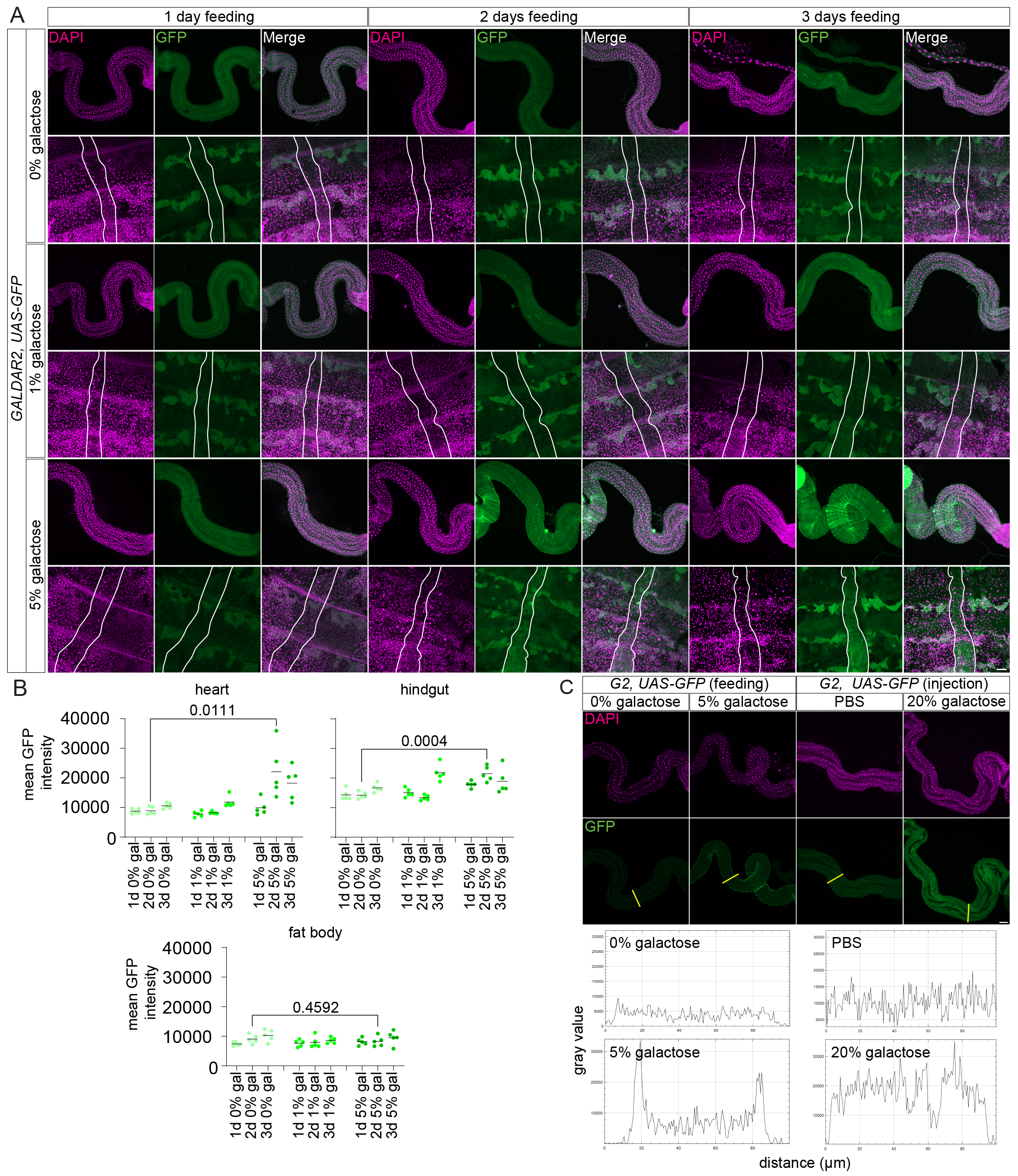

Supplement: S2 Fig — (A) Stacked confocal images of hindguts and abdomens (heart: between white lines) of adult virgin female G2, UAS-GFP flies with 1 day (left column), 2 days (middle column), and 3 days (right column) feeding with 0% (top row), 1% (middle row), or 5% (bottom row) galactose. (B) Quantification of mean GFP fluorescence intensities of heart, hindgut, and fat body shown in (A). Black lines show the mean value. Two-tailed unpaired t test. (C) Single confocal images of adult virgin female G2, UAS-GFP flies 3 days after feeding (left) and 1 day after 20% galactose injection (right). Fluorescence intensity profiles of yellow lines indicated on the images (bottom), showing GFP signals in visceral muscles and ECs in hindguts after feeding and injection (DAPI in magenta, GFP in green). S1 Data provides the source data used for all graphs and statistical analyses. Scale bars, 50 μm in all figures. (TIF) [file pbio.3002549.s002.tif]

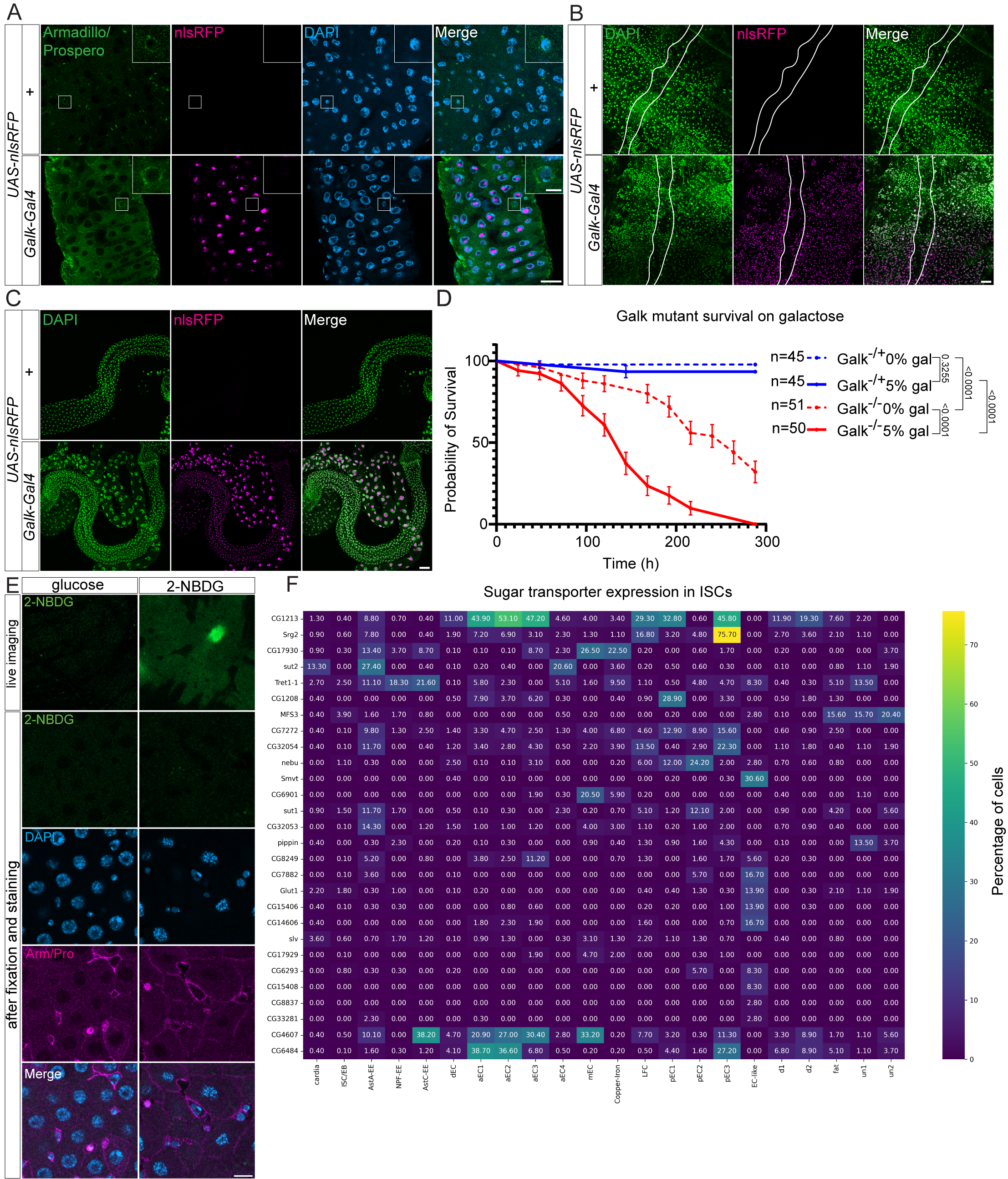

Supplement: S3 Fig — (A) Confocal images of R2 regions of virgin adult female midguts showing UAS-nlsRFP only (top) or Galk-Gal4, UAS-nlsRFP (bottom) expression. ISCs are highlighted in small panels. (B) Stacked confocal images of virgin adult female abdomen, showing UAS-nlsRFP only (top) or Galk-Gal4, UAS-nlsRFP (bottom) expression in fat body, oenocytes, and heart muscle (between white lines). (C) Stacked confocal images of virgin adult female hindguts, showing UAS-nlsRFP only (top) or Galk-Gal4, UAS-nlsRFP (bottom) expression. (D) Survival of adult male Galk-/- and Galk-/+ flies on 0% and 5% galactose supplemented food. Logrank (Mantel–Cox) test. (E) Confocal images of same midguts in live (top) and fixed condition (2-NBDG in green, DAPI in blue, Armadillo/Prospero in magenta). (F) Heatmap showing percentage of cells with detected expression of sugar transporters in cell clusters in Drosophila midgut according to single-cell RNA sequencing data. Note the lack of expression in ISC/EB.Data are representative of at least 3 independent experiments. S1 Data provides the source data used for all graphs and statistical analyses. Scale bars, 20 μm in large, 5 μm in small panels in (C), 50 μm in (D) and (E). (TIF) [file pbio.3002549.s003.tif]

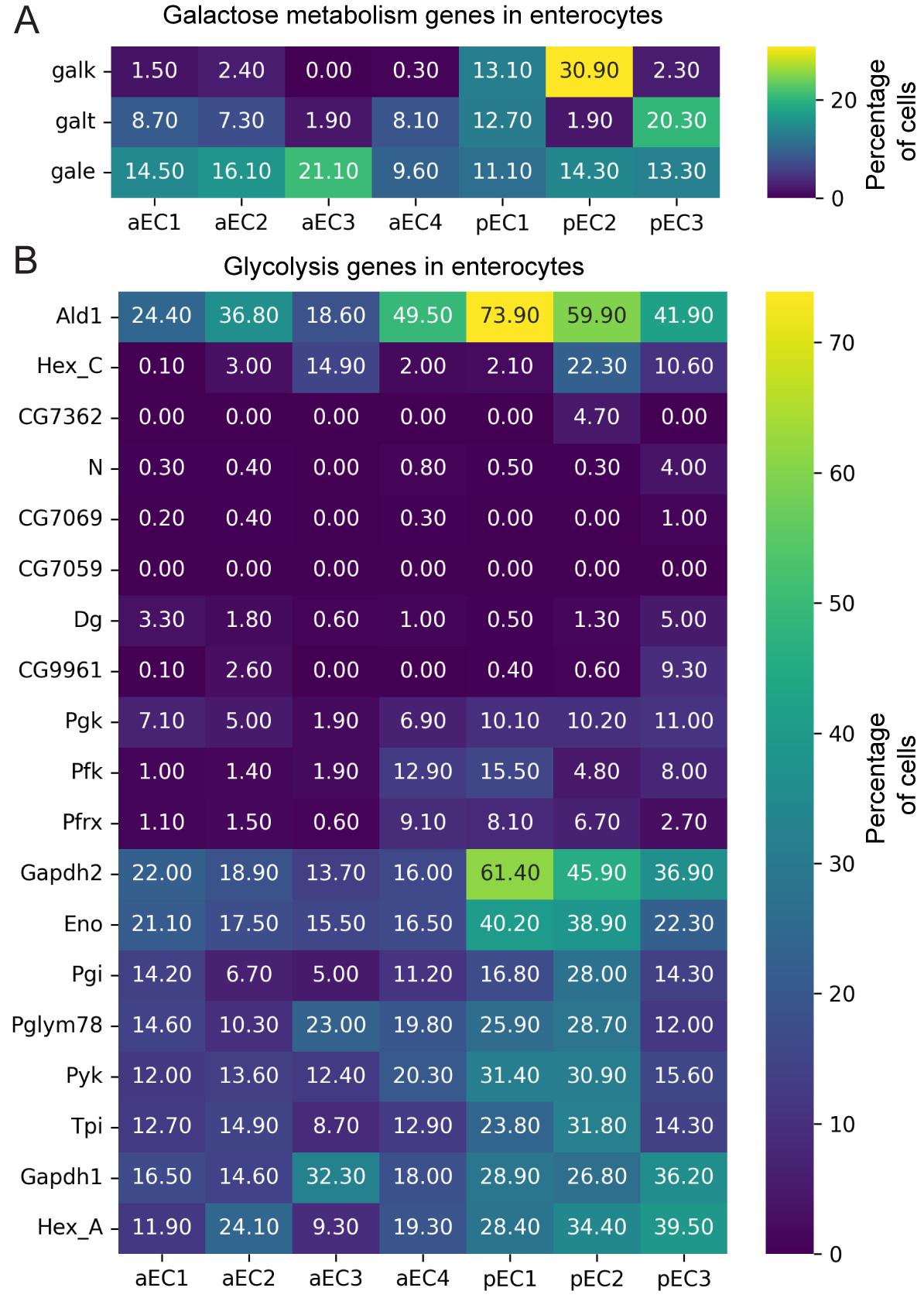

Supplement: S4 Fig — (A) Heatmap showing percentage of cells with detected expression of galactose metabolism genes in enterocytes according to single-cell RNA sequencing data (aEC and pEC indicate ECs in the anterior and posterior parts, respectively). (B) Heatmap showing percentage of cells with detected expression of glycolysis genes in enterocytes according to single-cell RNA sequencing data (aEC and pEC indicate ECs in the anterior and posterior parts, respectively). (TIF) [file pbio.3002549.s004.tif]
