# Supplementary material for: GALDAR: A genetically encoded galactose sensor for visualizing sugar metabolism in vivo
Source: PLoS Biol. 2024 Mar 19;22(3):e3002549. doi: 10.1371/journal.pbio.3002549 (PMC10950222; doi:10.1371/journal.pbio.3002549)
Supplement: Attachment — Submitted filename: GALDAR paper rebuttal checked.pdf [file pbio.3002549.s006.pdf]

We thank the editor and all reviewers for their helpful comments. We addressed almost all comments by experimenting. Because of their insightful comments, the manuscript improved tremendously. To address the raised concerns, we decided to complete development of a newer version of our sensor, which we were working on as another research project, and to include it in this manuscript. We believe with this technical improvement, our sensor will be more beneficial for scientists working on sugar metabolism in *Drosophila*. We also emphasized physiological relevance of galactose metabolism more in revised text and included data with the natural fig fruit. We hope that the editors and the reviewers will be satisfied with our revised manuscript. Following is a point-by-point response.

Reviewer #1: This manuscript describes a transcriptional galactose sensor in Drosophila, called GALDAR. It is based on yeast galactose sensing system, composed of transcriptional activator GAL4, its inhibitor GAL80 as well as GAL3, a galactose responsive inhibitor of GAL80. When animals are fed with high galactose diet, the sensor gets activated in the enterocytes and some enteroendocrine cells of anterior midgut. In addition to galactose feeding, the sensor is tested by galactose injections, which lead to activation of the sensor in some, but not all tissues. The authors hypothesize that limited sensor activation in some tissues is due to active galactose metabolism. This seems to be that case, as strong ubiquitous sensor activation is observed in galactokinase (Galk) mutants with defective galactose catabolism. Despite this, intestinal stem cells (ISCs) do not display any sensor activation, implying that ISCs do not take up galactose. As galactose is expected to be transported by the same transporter as glucose, the authors suggest that ISCs also lack glycose uptake. This is confirmed by using and established fluorescent glucose analogue, 2-NBDG, which is also excluded from the ISCs. Finally, the sensor is tested in the context of larval development. The sensor is activated by galactose in enterocytes and epidermis of L2 and L3 larvae. However, in the fat body the sensor is only active in L2, but not in L3. This is correlated with elevated expression of galactokinase in the fat body during L3, suggesting that sensor is inactivated during development because of elevated galactose catabolism.

In sum, the characterization of the sensor is technically sufficient and convincing evidence is presented that the sensor reports high intracellular galactose levels in vivo, in particular in tissues with slow galactose catabolism. In addition, the validation of the sensor leads to two potentially interesting observations, i.e. the lack of galactose/glucose import in ISCs and developmental changes in fat body galactose metabolism. However, the physiological roles of these findings are not studied further.

We gratefully appreciate the reviewer's positive appreciation of our galactose sensor.

Despite these strengths, I am not convinced that this manuscript represents such a significant advance in the field that it warrants to be published in a selective broad audience journal, like PLoS Biology. My main reservations are related to:

1. Physiological importance of Drosophila galactose metabolism: The sensor can certainly be a useful tool in analyzing galactose metabolism in the fly. However, the introduction of the manuscript did not really highlight any biological process that would specifically depend on galactose metabolism. The fact that some fruits eaten by Drosophila contain some galactose implies that the animal indeed needs a capacity to catabolize galactose, but whether galactose metabolism specifically influences some processes with broader physiological impact is not outlined in the introduction or demonstrated by the study.

We thank the reviewer for pointing this out and apologize for our failure to sufficiently emphasize the relevance of galactose metabolism in Drosophila. We believe galactose metabolism is very relevant for flies as well as humans. First, genes regulating galactose metabolism such as GALT, GALE and GALK are all conserved in Drosophila. Mutants of these genes manifest various phenotypes such as shortened lifespan, climbing defects and fecundity defects. Importantly, these mutants also demonstrate unhealthy phenotypes even without addition of extra galactose. We emphasized these aspects in introduction as follows:

Although the lab's standard fly food does not contain much galactose, the fly's ancestral fruit, marula contains galactose [1, 2] and many fruits and vegetables contain both free and bound galactose [3-6] , which suggests that galactose is a physiologically relevant sugar for Drosophila. In fact, adult flies can keep living with only galactose (Fig. S1A), indicating that Drosophila uses galactose as an energy source. Galactose metabolism genes such as galactokinase, GALT (Galactose-1p uridyltransferase) and GALE (UDPgal 4'-epimerase) are phylogenetically conserved, including Drosophila [7]. Importantly, mutants of these enzymes accumulate galactose-derived metabolites and demonstrate shortened lifespan, the lower climbing ability and a fecundity defect, which are aggravated by addition of extra galactose [7-13].

Besides citing the literature, we also demonstrated relevance of galactose metabolism in flies ourselves (Fig S3D). We experimentally investigated survivability of the Galk null mutant and heterozygous control flies when challenged with 5% galactose. Galk null flies died out on the 12th day when challenged with 5% galactose whereas galactose

did not have any effect on survival of control flies. We also observed that the life span of Galk null flies was shorter than control flies even when not challenged with extra galactose, implying that the very small amount of galactose in yeast affects fitness of the Galk mutant. These results are similar to a previous galactosemia model generated by knocking-out Galt, another galactose catabolism gene downstream of Galk in Drosophila [12]. Although we did not include the data in the manuscript, we also observed many eggs of Galk null parent flies did not hatch 1 day after egg laying. Indeed, a previous study found a decrease in fecundity in the flies with deteriorated galactose metabolism when compared to wild type flies even in the absence of extra galactose, highlighting the importance of galactose metabolism [13].

In addition to the phenotypes of the genes that regulate galactose metabolism, we also added data that GALDAR3 detects physiological levels of galactose in natural fig fruits in this revised paper (Please see below).

We humbly speculate that the apparent perception that galactose may not be very relevant for flies might be coming from our common practice to use the artificial lab food that is mainly composed of yeast and glucose/sucrose. However, we would like to emphasize that, in the wild, flies eat more diverse foods, which often contain galactose: fruits include galactose, and the wild yeast also uses galactose as an energy source, which is exemplified by its Gal system itself. These strongly suggest that galactose is abundant in the wild, and a natural, relevant sugar for organisms in the wild. Again, this is strongly supported by the conservation of galactose metabolism genes throughout species, including Drosophila, and that their mutants are susceptible to galactose. We hope that our paper demonstrates the importance and relevance of galactose metabolism in flies, which may have been less appreciated in the lab's environment.

2. Practical applicability: Some features of the sensor significantly limit its usefulness.
C) The galactose sensor is based on the activity of GAL4 transcription factor, which is the most widely used expression system in Drosophila. Therefore, the sensor cannot be readily used in combination with the most abundant RNAi or transgenic tools under the control of UAS. Instead, for genetic experiments it needs to be combined with mutants or with less widely used heterologous expression systems, like LexA/Aop.

We want to address this point first since it is relevant to other points. Many thanks for pointing out this important limitation of GALDAR1 and 2. We appreciate this reviewer's deep understanding of our GALDAR system. We were aware of this limitation of GALDAR1 and GALDAR2 and have been working on another version of GALDAR (we call GALDAR3) for possibly another publication. Upon this rightful concern of the

reviewer, however, we decided to complete development of the new sensor and to add it as a new figure to the manuscript. The design of GALDAR3 circumvents almost all drawbacks of the previous designs. We introduced the following features to make GALDAR independent of Gal4-UAS system (Fig 6A):

1. We fused Gal80 to GFP.
2. We introduced a mutation in Gal80 (G310D) that inhibits binding to Gal4 but not to Gal3 [14].
3. We enforced Gal3 to localize to the nucleus by adding NLS (nuclear localization signal) to Gal3.

The sensor is ubiquitously expressed using a tubulin enhancer on a single transcript, which after translation cleaves into two proteins via a P2A peptide. With these changes, upon an increase in the intracellular galactose levels, nls-Gal3 interacts with and traps Gal80(G310D)-GFP in the nucleus, increasing nuclear GFP intensity.

Using GALDAR3, it is possible to express the desired gene or RNAi using available Gal4/UAS lines. We demonstrate this by combining GALDAR3 with Myo1D-Gal4, UAS-CD8-RFP (Fig 6D-E). GALDAR3 shows a clear increase in the nuclear GFP levels depending on ingested galactose concentration while allowing for CD8-RFP to be expressed using enterocyte specific Gal4 driver.

GALDAR3 is a transcription-independent biosensor, which in general provides better temporal resolution with a higher sensitivity. However, we also think GALDAR1/2 has some merits as a transcription-dependent system: it enables gene manipulation based on signals and can capture long-term, accumulating effects of galactose. For example, the regional difference of signals in the gut was more pronounced with GALDAR1/2 than GALDAR3 likely due to signal saturation with GALDAR3. We included this information in the revised manuscript.

A) The manuscript shows that the sensor activity can only be detected under conditions of relatively high galactose availability introduced by by feeding/injection. How these galactose levels relate to physiological galactose intake is unclear.

This is again a very insightful comment. Some of the fruits which are possibly relevant to the Drosophila diet contain relatively high concentrations of galactose. Marula tree gum is reported to have a high amount of galactose [1, 2] implying the fruit might also be a natural source of galactose for Drosophila. Unfortunately, we could not get our hands on marula to test our sensor on, due to Japan's strict regulations on fruit import. Another fruit possibly relevant to the Drosophila diet in the wild, the fig fruit contains a relatively high amount of free galactose [5, 6]. GALDAR3 was able to reflect the

galactose concentration in the fig fruit as an increase in nuclear GFP levels in the enterocytes of the midgut as well as adipose cells in the fat body. The amount of galactose levels in fig naturally correspond to physiological intake.

B) The most important limitation is that the galactose sensor activity depends on galactose catabolism (sensor activity greatly enhanced by loss of Galk). Thus, no sensor activity is detected in tissues with high galactose catabolism (perhaps most physiologically relevant?).

We interpret that the reviewer's concern is that GALDAR1/2 has slow kinetics, which makes the temporal resolution and the sensitivity low, although we humbly think the phenomenon itself that galactose catabolism affects galactose concentrations is a logical consequence.

As mentioned above, GALDAR3 is a more sensitive probe than GALDAR1/2, since GALDAR3 does not require time for transcription, translation and maturation of a fluorescent protein for detection of intracellular galactose. The sensor is readily transcribed and translated in all cells. A similar experiment conducted in yeast suggests the interaction between nls-Gal3 and Gal80 happens virtually instantly upon addition of galactose to the medium [14]. This indicates that GALDAR3 is relatively faster and more sensitive than previous versions and it is possibly less affected by the galactose metabolism, demonstrated by increased nuclear GFP signals even in tissues with relatively high galactose metabolism such as the fat body (Fig 6B-C). Although GALDAR3 is more sensitive, it lacks the flexibility and gene induction ability of GALDAR1 and GALDAR2. Additionally, signals with GALDAR3 could be easily saturated, which occurs in the gut where regional differences of galactose concentrations were more detectable with GALDAR1/2. Thus, each GALDAR version has its own uses.

D) The authors suggest alternative uses for GALDAR, such as measuring glucose uptake (shared transporters of glucose and galactose) and the use of GALDAR as an inducible expression system. These might broaden the usability of the tool, but it remains to be demonstrated whether GALDAR is able to outperform the existing methodologies, such as 2-NBDG and GeneSwitch, respectively.

GALDAR1/2 can be used to drive expression of a gene of interest in the tissues which incorporate galactose. By design, the induction reagents and the genetic setups are different, thus a comparison of GALDAR1/2 and GeneSwitch is practically difficult. We

only note that GeneSwitch by RU486 has some issues such as leaky expression and effects on mitochondrial gene expression [15, 16]

Regarding 2-NBDG, we propose the following points make GALDAR superior to 2-NBDG:
- 2-NBDG is water-soluble and not retained in the cells after washes in the immunohistochemistry process [17], whereas all GALDAR versions are fixable, which makes combinations with IHC possible. In addition to available literature, we executed experiments and demonstrated this by ourselves using midgut enterocytes by live imaging and consequent fixation and IHC on the same tissue (Fig S3E).
- The fluorescence of 2-NBDG is not stable and decreases rapidly once inside the cell [18] whereas fluorescent proteins retain fluorescence for longer time periods.
- 2-NBDG is relatively expensive which limits its accessibility.
- Excitation and emission maxima of 2-NBDG coincides with GFP, hindering its usage with the commercially available GFP-tagged proteins. GALDAR1/2 are flexible and readout fluorescent protein can be swapped with any desired one, as demonstrated in the manuscript with GFP and nls-RFP.

3. New biological insight: One possible way to increase the general importance of this study (and simultaneously reduce the concern on limited physiological role of galactose, see point #1) would be to use GALDAR to produce data that brings new biological insight into galactose metabolism in vivo. The finding that galactose catabolism in the fat body is regulated during larval development is a potentially interesting lead, but its importance remains unclear.

We sincerely agree with the reviewer that this larval transition in galactose metabolism is potentially interesting and further studies should reveal more insights for the sugar metabolism. However, we want to limit this manuscript for the introduction of GALDAR as a new tool and consider deep mechanistic pursuit as out of scope of this method paper. However, in this revised manuscript, we newly measured galactose using LC-MS/MS (Fig 5E) and found that the galactose concentrations correlate with Galk expression in a reverse manner during development.

In addition to these major concerns, I noticed statements that are misleading:

1. Intruduction states: "For example, lactate was recently discovered as an important circulating sugar, contrary to the long-held belief that glucose is the important circulating sugar."

Lactate is not a sugar, it is an anion of an organic acid. It is a metabolite of sugars, but also of other biomolecules, such as amino acids. It is well known that, in addition to glucose, there are several metabolites that are present in high levels in circulation and can be catabolized by cells to release energy. Lactate is one such molecule, but also certain amino acids (such as glutamine) and ketone bodies serve as circulating energy source. Moreover, it is well established that the most abundant circulating sugar in Drosophila is not glucose, but trehalose.

We thank the reviewer for pointing out this mistake. The text was modified.

2. The Introduction states: "We focused on galactose, which is particularly important for animal development at the early stage." This is a very unspecific statement. Does early stage development refer to embryonic development or young mammals feeding on milk? Is galactose important also for early stage development of Drosophila?

We agree with the reviewer that the text was not clear about the importance of galactose in development. The text was modified.

3. The GALDAR sensor gets activated in the enterocytes of anterior, but not posterior, midgut. Based on that observation, the following conclusions are made in the Results:
"These observations suggest that enterocytes in the anterior midgut absorb galactose and transfer it to the visceral muscle and likely to the hemolymph (Fig 1I)"
"These observations suggest a possible scenario where enterocytes in the anterior midgut uptake galactose and transfer it to the visceral muscles and the hemolymph, followed by consumption in the hindgut visceral muscles and the heart muscles."

The data produced later on in Figure 3 shows that the whole midgut takes up galactose, but the sensor is not activated in the posterior due to rapid elimination. Therefore the conclusions about anterior midgut being the site of galactose absorption are misleading, as subsequent experiments show that this is not the case.

We agree with the reviewer's rightful comment. Accordingly, we modified the text, by removing our original overinterpretation.

Reviewer #2: In this work, the authors developed a genetically encoded sensor called GALDAR by transgenically implementing the galactose sensing system from yeast into Drosophila. While this demonstration is interesting, multiple issues decrease my enthusiasm. The following comments should be addressed before considering its publication in this journal.

1. The manuscript should be carefully and extensively revised in terms of flow, structure, and grammar. For example, but not limited to this, the flow of the abstract is not smooth with disconnected statements.

We thank this reviewer's useful advice. We made our best effort to make the flow of the text better in this revised manuscript.

2. Some data or figures are missing statistical analyses with associated p-values and others. For proper comparison between data, all datasets should have statistical analysis results.

We added statistical analyses to relevant data in the revised manuscript.

3. As a method or resource paper, their article would have a bigger impact on the community if the authors show the approach's generalizability or multiple applications to answer biological questions. This issue should be addressed to make the article more useful.

Although we consider that this paper is a report of a new genetic tool, we also provided some biological insights such as almost no detectable galactose uptake in ISCs and the developmental transition of galactose metabolism. We further extended discussion on potential applications of GALDAR in the revised manuscript.

4. The authors have used qPCR analyses for key experiments, but it seems that qPCR data reporting does not follow generally accepted guidelines (i.e., MIQE). This issue should be addressed.

We followed the reviewer's useful advice.

5. Sometimes, figure texts are too small to be legible.

We apologize for this mistake. We tried to make all letters legible in the revised figures.

6. The method section needs more details to allow other researchers to reproduce the authors' experiments.

We added more details to the methods in the revised text.

Reviewer #3: In this well-written manuscript by Sakizli and Yoo, the authors have engineered a galactose sensing system in Drosophila. While the yeast proteins Gal4 and Gal80 are commonly used as tools by the Drosophila genetics community, the third component of yeast galactose sensing system, Gal3, has not previously been used in the fly. Here the authors drive expression of all three Gal proteins in the fly, thus establishing the first Drosophila galactose sensor system (referred to as GALDAR). The authors then use multiple methods to demonstrate that the system responses to changes in galactose abundance. As an extension of their studies, they also examine activation of GALDAR in a variety of tissues, including the intestine. Surprisingly, their study reveals that galactose, and by extension glucose, is not readily transported into the intestinal stems cells. This later observation is quite important and warrants further investigation.

Overall, the study is well executed, and the sensor will likely be of broad interest to the Drosophila community (especially since the system can be potentially used to temporally control UAS- transgene expression). Moreover, the observation that intestinal stem cells do not import galactose, or glucose, at detectable levels is of great importance. However, there are a few issues that the authors need to address:

1. The authors should add a key negative control experiment to demonstrate that GALDAR does not response to high levels of glucose. As the authors mentioned, glucose and galactose are very similar (both monosaccharides use the same transporter), yet glucose is never used as a negative control experiments. I realize that in most experiments, the authors used a sucrose control, and sucrose is composed of glucose and fructose. However, the monosaccharide glucose is the more appropriate control and I'm confused as to why sucrose was used instead. To address this issue, I'd ask that the authors quantify tissue-specific GALDAR activity in adult flies where glucose was injected into the hemocoel.

This is a rightful comment. Although the specificity of the Gal3 system has been shown in yeast, we failed to demonstrate it in flies. To experimentally address the reviewer's comment, we demonstrated that feeding 5% sucrose, 5% glucose or 30% glucose for 3 days does not activate GALDAR2 whereas 5% galactose does (Fig S1E-F).

2. The authors repeatedly state that galactose and glucose do not enter ISCs. I do not believe that their methods are sensitive enough to make this statement. While I agree that glucose/galactose transport is significantly lower in ISCs compared with other intestinal cells, there is very likely some level of glucose/galactose transport in these cells. Either the authors must use stable isotope tracers to demonstrate that glucose

doesn't enter the ISCs (an exceptionally difficult experiment), or they need to edit the text in a manner that provides the reader with a more nuanced view. In addition, the authors should determine if the expression of genes that encode glucose transporters are reduced in ISCs - for this point, the authors can simply use preexisting data from the Fly Cell Atlas and do not need to conduct new experiments.

We agree with the reviewer. We cannot completely say ISCs never uptake glucose/galactose. Thus, we changed the text as follows: we discovered intestinal stem cells do not uptake detectable levels of galactose or glucose. This emphasizes that the galactose amount is just below the detectable levels of sensors.

Regarding sugar transporter expression in ISCs, we thank the reviewer's useful advice. We checked the Fly Cell Atlas and indeed ISCs have very low expression of sugar transporters. We included the data in Fig S3F.

3. I'm quite interested by the observation that GALDAR signal decreased during the L2 to L3 larvae while Galk expression increased during this time, suggesting that galactose metabolism is regulated in response to a developmental timing mechansims (very cool). I'd ask that the authors determine if this change significantly alters the larval galactose pool by directly measuring galactose levels in L2 vs L3 larvae.

We are thankful for the reviewer's enthusiasm with our findings. In order to answer this point, we measured galactose amounts in wild type OregonR L2 and L3 larvae by LC-MS/MS. Even without extra galactose addition, we were able to detect endogenous galactose in L2 larvae, which became almost undetectable in L3 larvae. When the larvae were grown in 5% galactose supplemented food, galactose amounts decreased significantly with development, in accordance to increased Galk expression.

I also have a few minor edits:

1. Figure 1S. The survival curve in panel A is very difficult to read. Please edit so that the reader can easily distinguish the line representing each treatment.

We thank the reviewer for this suggestion. We modified the figure in the revised manuscript.

2. Figure 3B. Note the location of the GALDAR2 animals fed glucose with either asterisks or arrows. The current panel looks like a completely blank square.

We thank the reviewer for another useful suggestion. The figures were updated such that all flies are outlined and easily visible.

1. Mansourian S, Enjin A, Jirle EV, Ramesh V, Rehermann G, Becher PG, et al. Wild African Drosophila melanogaster Are Seasonal Specialists on Marula Fruit. Curr Biol. 2018;28(24):3960-8 e3. Epub 20181206. doi: 10.1016/j.cub.2018.10.033. PubMed PMID: 30528579; PubMed Central PMCID: PMCPMC7065024.
2. Leakey RRB. Potential for novel food products from agroforestry trees: a review. Food Chem. 1999;66(1):1-14. doi: Doi 10.1016/S0308-8146(98)00072-7. PubMed PMID: WOS:000079904500001.
3. Smith BG, Harris PJ. Polysaccharide Composition of Unlignified Cell-Walls of Pineapple [Ananas-Comosus (L) Merr] Fruit. Plant Physiol. 1995;107(4):1399-409. doi: DOI 10.1104/pp.107.4.1399. PubMed PMID: WOS:A1995QT40200042.
4. Acosta PB, Gross KC. Hidden Sources of Galactose in the Environment. Eur J Pediatr. 1995;154(7):S87-S92. doi: Doi 10.1007/Bf02143811. PubMed PMID: WOS:A1995RG85200019.
5. Owino W O, Nakano R, Kubo Y, Inaba A. Alteration in cell wall polysaccharides during ripening in distinct anatomical tissue regions of the fig (Ficus carica L.) fruit. 園芸学会雑誌 別冊, 園芸学会大会研究発表. 2003;72(2):504.
6. Ersoy N, Gozlekcki S, Kaynak L, editors. CHANGES IN SUGAR CONTENTS OF FIG FRUIT (FICUS CARICA L. CV. BURSA SIYAHI) DURING DEVELOPMENT2007.
7. Daenzer JM, Fridovich-Keil JL. Drosophila melanogaster Models of Galactosemia. Curr Top Dev Biol. 2017;121:377-95. Epub 20160803. doi: 10.1016/bs.ctdb.2016.07.009. PubMed PMID: 28057307; PubMed Central PMCID: PMCPMC5683716.
8. Daenzer JM, Sanders RD, Hang D, Fridovich-Keil JL. UDP-galactose 4'-epimerase activities toward UDP-Gal and UDP-GalNAc play different roles in the development of Drosophila melanogaster. PLoS Genet. 2012;8(5):e1002721. Epub 20120524. doi: 10.1371/journal.pgen.1002721. PubMed PMID: 22654673; PubMed Central PMCID: PMCPMC3359975.
9. Jumbo-Lucioni P, Parkinson W, Broadie K. Overelaborated synaptic architecture and reduced synaptomatrix glycosylation in a Drosophila classic galactosemia disease model. Dis Model Mech. 2014;7(12):1365-78. Epub 20141017. doi: 10.1242/dmm.017137. PubMed PMID: 25326312; PubMed Central PMCID: PMCPMC4257005.
10. Lee TE, Yu L, Wolf MJ, Rockman HA. Galactokinase is a novel modifier of calcineurin-induced cardiomyopathy in Drosophila. Genetics. 2014;198(2):591-603. Epub 20140731. doi: 10.1534/genetics.114.166777. PubMed PMID: 25081566; PubMed Central PMCID: PMCPMC4196615.
11. Sanders RD, Sefton JM, Moberg KH, Fridovich-Keil JL. UDP-galactose 4' epimerase (GALE) is essential for development of Drosophila melanogaster. Dis Model Mech. 2010;3(9-10):628-38. Epub 20100602. doi: 10.1242/dmm.005058. PubMed PMID: 20519568; PubMed Central PMCID: PMCPMC2931539.
12. Kushner RF, Ryan EL, Sefton JM, Sanders RD, Lucioni PJ, Moberg KH, et al. A Drosophila melanogaster model of classic galactosemia. Dis Model Mech. 2010;3(9-10):618-27. Epub 20100602. doi: 10.1242/dmm.005041. PubMed PMID: 20519569; PubMed Central PMCID: PMCPMC2931538.
13. Daenzer JM, Jumbo-Lucioni PP, Hopson ML, Garza KR, Ryan EL, Fridovich-Keil JL. Acute and long-term outcomes in a Drosophila melanogaster model of classic galactosemia occur independently of galactose-1-phosphate accumulation. Dis Model Mech.

2016;9(11):1375-82. Epub 20160824. doi: 10.1242/dmm.022988. PubMed PMID: 27562100; PubMed Central PMCID: PMCPMC5117221.
14. Pilauri V, Bewley M, Diep C, Hopper J. Gal80 dimerization and the yeast GAL gene switch. Genetics. 2005;169(4):1903-14. Epub 20050203. doi: 10.1534/genetics.104.036723. PubMed PMID: 15695361; PubMed Central PMCID: PMCPMC1449595.
15. Robles-Murguia M, Hunt LC, Finkelstein D, Fan Y, Demontis F. Tissue-specific alteration of gene expression and function by RU486 and the GeneSwitch system. NPJ Aging Mech Dis. 2019;5:6. Epub 20190521. doi: 10.1038/s41514-019-0036-8. PubMed PMID: 31123597; PubMed Central PMCID: PMCPMC6529481.
16. Scialo F, Sriram A, Stefanatos R, Sanz A. Practical Recommendations for the Use of the GeneSwitch Gal4 System to Knock-Down Genes in Drosophila melanogaster. PLoS One. 2016;11(8):e0161817. Epub 20160829. doi: 10.1371/journal.pone.0161817. PubMed PMID: 27570965; PubMed Central PMCID: PMCPMC5003377.
17. Itoh Y, Abe T, Takaoka R, Tanahashi N. Fluorometric determination of glucose utilization in neurons in vitro and in vivo. J Cereb Blood Flow Metab. 2004;24(9):993-1003. doi: 10.1097/01.WCB.0000127661.07591.DE. PubMed PMID: 15356420.
18. Kao TY, Wu HW, Lee SS, Liang PH, Guh JH, Hsu LC. Characterization of a fluorescent glucose derivative 1-NBDG and its application in the identification of natural SGLT1/2 inhibitors. J Food Drug Anal. 2021;29(3):521-32. Epub 20210915. doi: 10.38212/2224-6614.3371. PubMed PMID: 35696243; PubMed Central PMCID: PMCPMC9261792.
