# Supplementary material for: GALDAR: A genetically encoded galactose sensor for visualizing sugar metabolism in vivo
Source: PLoS Biol. 2024 Mar 19;22(3):e3002549. doi: 10.1371/journal.pbio.3002549 (PMC10950222; doi:10.1371/journal.pbio.3002549)
Supplement: Attachment — Submitted filename: response to reviewers.docx [file pbio.3002549.s008.docx]

We addressed all of the remaining comments by Reviewer 1 as follows.

1. The text has been somewhat improved and the erroneous statements pointed out earlier have been eliminated. There are still many paragraphs where the quality of writing is below the standard usually seen in journals like Plos Biology.  Some examples:

-"…the fly's ancestral fruit, marula contains galactose…"

Text was modified as follows.

*“Although the lab’s standard fly food does not contain much galactose, marula, an African fruit that is the ancestral host of D. melanogaster contains galactose”*

-"We heterologously transplanted yeast's galactose sensing system into Drosophila. Yeast senses galactose using Gal4, Gal80 and Gal3. …. We implanted yeast's whole Gal system to Drosophila and generated a heterologous biosensor for galactose. …. We transgenically incorporated the yeast galactose metabolism genes, including Gal3, Gal4 and Gal80…"

Text was modified as follows.

*“We heterologously transplanted the galactose sensing system of yeast into Drosophila.* *Yeast detects galactose using Gal4, Gal80 and Gal3. In absence of galactose, Gal80 binds to the transcriptional activator Gal4 and inhibits transcription of genes with a UAS sequence [24, 25]. Gal4 sequestration by Gal80 is lifted upon a conformational change of galactose-bound Gal3, which has a high affinity to Gal80, allowing the transcriptional machinery to be recruited by free Gal4 and consequent transcription [24, 25]. The Perrimon group introduced the Gal4-UAS system to Drosophila [26], followed by addition of Gal80 by the Luo group [27]. However, galactose sensing Gal3 has never been incorporated to Drosophila.* *We implanted the whole Gal system of yeast to Drosophila and generated a heterologous biosensor for galactose.”*

"We have thus far detected galactose by GALDAR in adult flies. We investigated galactose metabolism during larval development. During larval development, there is an ecdysteroid-mediated dynamic change of sugar metabolism [35-37]. GALDAR larvae were developed with 0% and 5% galactose (Fig. 5A)…"

Text was modified as follows.

*“Thus far, we have detected galactose by GALDAR in adult flies. We investigated galactose metabolism during larval development. During larval development, there is an ecdysteroid-mediated dynamic change of sugar metabolism [34-36]. GALDAR larvae were cultivated under distinct galactose concentrations, specifically 0% and 5% (Fig. 5A).”*

Notably, the new text included in the end of the Results section to describe GALDAR3 is written in a more proficient way. The rest of the text should be carefully edited to meet the same standards.

2. I earlier made a comment about the detected GALDAR sensor activity in the anterior midgut, which does not seem to reflect high, but rather low, galactose metabolism in the anterior midgut (leading to galactose accumulation). This is because inhibition of galactose metabolism increases the signal in the posterior part. Now the authors state the following (in two separate parts):

"We observed that the anterior midgut (R2 region) of adult virgin females exhibits GFP signals with a presence of galactose (Fig 1D). This observation is reminiscent of the previous reports showing that carbohydrate metabolism is compartmentalized in the anterior midgut of female Drosophila [31, 32]."

"On the Galk mutant background, most tissues and cell types were GALDAR positive after 2-day feeding with 2% galactose (Fig 4B). This suggests that galactose is efficiently metabolized in most tissues at a relatively high rate, which affects the intracellular concentrations of galactose detected by GALDAR. Consistently, the whole adult midgut exhibits GALDAR signals after feeding 2% galactose with the Galk mutant background as opposed to the restricted GALDAR signals in anterior midgut with 5% galactose feeding in the wildtype background (Fig 4C)."

The data shown in Figure 1 (when considered alone) does indeed suggest that also galactose metabolism occurs in the anterior parts. However, when taken into account the data in figure 4, the posterior part seems in fact to be faster in catabolizing Galactose than the anterior part. This is in contrast to the earlier findings (on anterior expression of carbohydrate metabolism genes). To clarify the issue, it would be advisable to relate the findings of this manuscript to the existing data in a more specific and explicit manner. Rather than looking at random carbohydrate metabolism genes unrelated to galactose, to use the existing published datasets and specifically analyze the expression of genes encoding galactose catabolizing enzymes in the anterior vs. posterior parts of the intestine. This would allow you to answer whether their regional expression corresponds to the data produced by the GALDAR sensor.

We incorporated this reviewer’s useful advice and analyzed expression of galactose metabolism genes and glycolysis genes (Fig S4). We discussed this and also compared our findings to the previous findings as follows in discussion.

*“In general, uptake and metabolism of galactose affect its intracellular concentration. G1/G2 labeled enterocytes in the anterior midgut while G1/G2 in the Galk mutant and G3 labeled enterocytes in both anterior and posterior parts of the midgut. Although clarification based on experimentation is necessary, this strongly suggests that the regional difference of G1/G2 signals stems from the galactose metabolism difference: the anterior region has slower metabolism and the posterior has faster one. Consistently, Fly Cell Atlas [33] indicates that enterocytes in the posterior region tend to have higher expression of genes related to galactose and glucose metabolism than in the anterior region (Fig S4A-B) while expression levels of sugar transporters are comparable in both regions. This is in a contrast with the previous findings of restricted expression of maltase and polysaccharide metabolism-related genes in the anterior midgut of female flies, indicating that each process of sugar metabolism is compartmentalized in different regions of the gut.”*
